# Intra-Species Variations of Bioactive Compounds of Two Dictyota Species from the Adriatic Sea: Antioxidant, Antimicrobial, Dermatological, Dietary, and Neuroprotective Potential

**DOI:** 10.3390/antiox12040857

**Published:** 2023-04-01

**Authors:** Ana Martić, Lara Čižmek, Nikolay V. Ul’yanovskii, Tina Paradžik, Lucija Perković, Gabrijela Matijević, Tamara Vujović, Marija Baković, Sanja Babić, Dmitry S. Kosyakov, Polonca Trebše, Rozelindra Čož-Rakovac

**Affiliations:** 1 Laboratory for Aquaculture Biotechnology, Division of Materials Chemistry, Ruđer Bošković Institute, Bijenička 54, 10000 Zagreb, Croatia; amartic@irb.hr (A.M.); perkovic@irb.hr (L.P.); gmatijev@irb.hr (G.M.); tvujovic@irb.hr (T.V.); mbakovic@irb.hr (M.B.); sanja.babic@irb.hr (S.B.); rozelindra.coz.rakovac@irb.hr (R.Č.-R.); 2 Center of Excellence for Marine Bioprospecting (BioProCro), Ruđer Bošković Institute, Bijenička 54, 10000 Zagreb, Croatia; 3 Laboratory of Natural Compounds Chemistry and Bioanalytics, Northern (Arctic) Federal University, Nab. Severnoy Dviny 17, 163002 Arkhangelsk, Russia; n.ulyanovsky@narfu.ru (N.V.U.); d.kosyakov@narfu.ru (D.S.K.); 4 Laboratory for Chemical and Biological Crystallography, Division of Physical Chemistry, Ruđer Bošković Institute, Bijenička 54, 10000 Zagreb, Croatia; tina.paradzik@irb.hr; 5 Faculty of Health Sciences, University of Ljubljana, Zdravstvena pot 5, 1000 Ljubljana, Slovenia; polonca.trebse@zf.uni-lj.si

**Keywords:** *Dictyota dichotoma*, *Dictyota fasciola*, brown seaweeds, chromatography, toxicity, proteins, carbohydrates, amino acids, bioactivity

## Abstract

The marine environment has a significant impact on life on Earth. Organisms residing in it are vital for the ecosystem but also serve as an inexhaustible source of biologically active compounds. Herein, the biodiversity of two brown seaweeds, *Dictyota dichotoma* and *Dictyota fasciola* from the Adriatic Sea, was evaluated. The aim of the study was the determination of differences in compound composition while comparing their activities, including antioxidant, antimicrobial, and enzyme inhibition, in connection to human digestion, dermatology, and neurological disorders. Chemical analysis revealed several terpenoids and steroids as dominant molecules, while fucoxanthin was the main identified pigment in both algae. *D. dichotoma* had higher protein, carbohydrate, and pigment content. Omega-6 and omega-3 fatty acids were identified, with the highest amount of dihomo-γ-linolenic acid and α-linolenic acid in *D. dichotoma.* Antimicrobial testing revealed a dose-dependent inhibitory activity of methanolic fraction against *Escherichia coli* and *Staphylococcus aureus*. Moderate antioxidant activity was observed for both algae fractions, while the dietary potential was high, especially for the *D. fasciola* dichloromethane fraction, with inhibition percentages of around 92% for α-amylase and 57% for pancreatic lipase at 0.25 mg/mL. These results suggest that Dictyota species might be a potent source of naturally derived agents for obesity and diabetes.

## 1. Introduction

Marine macroalgae are aquatic organisms divided into three groups: green, brown, and red, based on their pigment composition [1]. Because of their harsh living environment, such as temperature, light, and salinity variations, along with possible microbial toxins present, marine macroalgae are extremely rich in different bioactive compounds [2,3,4]. Over the past decade, there has been an increasing focus on the isolation and identification of bioactive compounds from brown macroalgae (Phaeophyceae). Many of these compounds, including specific nutrients and phytochemicals, are already recognized for their potential medicinal properties in the treatment and prevention of various diseases [4,5]. Polysaccharides produced by brown seaweeds, including alginates, fucoidans, and laminarins, have been found to possess various health benefits. Alginates have been associated with regulating appetite and promoting positive effects in the gastrointestinal tract, as well as exhibiting antidiabetic and antihypertensive effects. Fucoidans, a type of polysaccharide characteristic of brown macroalgae, have been linked to anti-inflammatory, antitumor, immunoregulatory, antioxidant, and antiviral activities. Laminarins are considered to be dietary fibers [6]. Dietary fibers may improve digestive health and have an important role in the prevention of several diseases (colorectal cancer, gastrointestinal inflammation, and others) [7]. It has been previously reported that brown macroalgae such as *Hizikia fusiforme*, *Undaria pinnatifida*, and *Laminaria* sp. contain high amounts of dietary fiber [8]. It is also reported that *Bifurcaria bifurcata*, *Halidrys siliquosa*, *Cystoseira tamariscifolia*, and *Desmarestia ligulata* exhibited cytotoxic activity in several cancer cell lines [3]. Genus Dictyota exhibits a significant range of bioactivity from antioxidant to anticancer. Some research points out *Dictyota dichotoma* as a macroalga with high anti-cancer potential [3,5]. Furthermore, phenolic compounds present in brown macroalgae (i.e., *Sargassum muticum*, *Euchema cottonii*, and *Ecklonia cava*) are considered to exhibit anti-inflammatory, antioxidant, and antidiabetic activities [4,7]. Brown seaweeds also contain a wide variety of pigments, including fucoxanthin, chlorophyll a, chlorophyll c1, chlorophyll c2, β-carotene, and violaxanthin [9]. Fucoxanthin is a secondary metabolite produced in the chloroplast of brown macroalgae with great biological activities and protective properties for human health [10]. It has earned attention due to its promising effects in terms of anti-obesity, antidiabetic, and antioxidant activities [11]. In recent years, it has been extensively explored and used for functional food, feed, cosmetics, and medicine [10]. Additionally, different amounts of proteins, fatty acids, vitamins, minerals, and secondary metabolites make brown macroalgae great candidates for the functional food market and application in diverse industries [4]. Nowadays, only nine brown macroalgal species, namely *Himanthalia elongate*, *Fucus serratus*, *F. vesiculosus*, *Ascophyllum nodosum*, *U. pinnatifida*, *L. japonica, L. saccharina*, *L. digitata*, and *Alaria esculenta*, are considered to be safe for human consumption [4].

The prevalence of modern-day diseases such as obesity, diabetes mellitus, Alzheimer’s, and Parkinson’s disease, is constantly increasing. Since some enzymes are included in different metabolic and degenerative disorders, their identification and activity determination are extremely important. One approach to treating the mentioned diseases involves the inhibition of the enzymes that play a role in their pathogenesis [12,13]. The enzymes α-amylase and pancreatic lipase are associated with human digestion and diseases connected with the digestive system. Most studies on the inhibition of enzymatic activity have primarily focused on phenolic and flavonoid compounds, as indicated by previous research [14]. However, recent studies have highlighted the potential of nutraceutical compounds and functional groups found in brown macroalgae, such as fucoxanthin, dieckol, and bromophenol, as promising inhibitors of α-amylase and pancreatic lipase [7,15]. Collagenase and tyrosinase belong to the group of enzymes associated with skin and skin-related diseases. Some researchers have suggested that fucoxanthin, terpenes, and phloroglucinols are compounds with anti-melanogenic effects [16]. On the other hand, acetylcholinesterase plays a significant role in neurodegenerative diseases (i.e., Alzheimer’s disease). In vitro studies revealed that aqueous-ethanol extracts rich in phenolic acids, phlorotannins, and flavonoids from macroalgae *Ulva lactuca, Gelidium pristoides, E. maxima,* and *Gracilaria gracilis* exhibit acetylcholinesterase-inhibitory activities [17]. While many studies have focused on investigating enzyme activity from plants, there is a limited number of studies on marine organisms, particularly macroalgae [18,19]. Therefore, there is a need for further research on the potential of marine seaweeds to inhibit enzymes that are involved in different pathogeneses. 

The abundance data of the Dictyota genus are mainly available for the coral reef and island communities in the northern Atlantic region, although they can be found all over the world [6]. However, their seasonality in abundance is highly dependent on the sea temperature. It has been previously reported that this genus tends to be present in the warm seas for most of the year, while it disappears during the hottest months. On the other hand, higher fertility and abundance were observed in the colder sea climates during the summer period but were absent in winter [6]. Except for abundance, the chemical composition of the samples and their biological activity depends on the harvesting season and the location of the collection [1]. In the literature, data regarding the application of bioactive compounds from the Dictyota genus were reported from the Red Sea [1,3] and the Aegean Sea [5] with observed differences in chemical composition and biological activities. However, sparse literature data regarding the *D. dichotoma* and *D. fasciola* chemical composition from the Adriatic Sea are available, with one study focusing on phenolic metabolites [20], while the other two focused on volatile organic compounds from *D. dichotoma* [21,22]. In this study, we investigated chemical composition, including amino acid, fatty acid, and pigment analysis in addition to antioxidant and antimicrobial activity, while reporting the in vitro enzyme inhibition activity of two brown macroalgae (*Dictyota dichotoma* and *Dictyota fasciola*). The aim was to define as many compounds as possible and correlate them with obtained activities. In addition, enzyme inhibition analysis, which included α-amylase and pancreatic lipase, involved in human digestion; tyrosinase and collagenase, associated with skin-related problems; and acetylcholinesterase, involved in neurological disorders, provided additional information on the therapeutic potential of Adriatic Sea organisms, particularly in relation to intra-species variations. 

## 2. Materials and Methods

### 2.1. Chemicals

The standards and chemicals purchased from Sigma-Aldrich (St. Louis, MO, USA) were L-ascorbic acid (≥99%), gallic acid (>97.5%), Trolox solution (6-hydro-2,5,7,8-tetramethylchroman-2-carboxylic acid, 97%), potassium sodium tartrate tetrahydrate (≥99%), TPTZ (2,4,6-tripyridyl-S-triazine, ≥98%), ABTS (diammonium salt of 2,2′-azino-bis(3-ethylbenzthiazolin-6-yl) sulfonic acid), AAPH (2,2-azobis (2-methylpropionamidine) dihydrochloride, 97%), dichloro-dihydro-fluorescein diacetate (≥97%, DCF-DA), fluorescein (free acid), sodium acetate (CH_3_COONa, ≥99%), copper (II) sulfate pentahydrate (CuSO_4_ × 5H_2_O, ≥98%), DPPH (2,2-diphenyl-1-picrylhydrazyl), sodium phosphate dibasic (Na_2_HPO_4_ ≥ 99.0%), sodium tetraborate decahydrate (Na_2_B_4_O_7_ × 10H_2_O, ≥99.5%), and formic acid (≥95%), as well as a collagenase activity colorimetric assay kit (Catalog No. MAK293), soluble starch, α-amylase from porcine pancreas (Type VI-B), acarbose (≥95%), iodine–potassium iodide solution, sodium dihydrogen phosphate anhydrous (H_2_NaO_4_P), 5,5′-Dithiobis-(2-Nitrobenzoic Acid) (DTNB, ≥98%), acetylcholin esterase (AChE, Type V-S), tacrin, acetylcholine iodide (ACTI, ≥97%), 4-Nitrophenyl palmitate, chloramphenicol (≥98%, HPLC), lipase from porcine pancreas, orlistat, and all chemicals used for the preparation of artificial water (AW): calcium chloride dihydrate (CaCl_2_ × 2H_2_O, ≥99%), magnesium sulfate heptahydrate (MgSO_4_ × 7H_2_O, ≥99.0%), sodium bicarbonate (NaHCO_3_, ≥99.7%), potassium chloride (KCl, 99.0–100.5%), and MeOH. The FAME Mix (C4–C24) standard (Supelco), pentadecane, 3,4-Dihydroxy-L-phenylalanine (L-DOPA, ≥98%, TLC), Kojic acid (≥99%), and tyrosinase from mushroom (lyophilized powder, ≥1000 unit/mg solid), amino acids standard (AAS 18) were also purchased from Sigma-Aldrich (St. Louis, MO, USA). Internal standards (L-norvaline and sarcosine), extended amino acids (L-asparagine, L-tryptophane, L-4-Hydroxyproline, L-glutamine), and FMOC and OPA reagents and borate buffer (0.4 M, pH 10.2) were purchased from Agilent (Santa Clara, CA, USA). 

The potassium persulfate (K_2_S_2_O_8_, >98%) was purchased from Scharlau (Regensburg, Germany), while iron (III) chloride (FeCl_3_, p.a.), sodium phosphate (Na_2_HPO_4_ × 2H_2_O, p.a.), monobasic sodium phosphate (NaH_2_PO_4_ × H_2_O, p.a.), methanol (p.a.), ethanol (p.a.), dimethyl sulfoxide (DMSO, p.a), sodium hydroxide (NaOH, p.a.), and hydrochloric acid (HCl, p.a.) were obtained from Kemika (Zagreb, Croatia) and hydrogen peroxide (H_2_O_2_, 30%) from Alkaloid Skopje (North Macedonia). All the solvents used for solid-phase extraction (SPE) and HPLC (methanol, isopropanol, acetonitrile, dichloromethane) and GC (n-hexane, chloroform) analysis were of HPLC grade and were purchased from Honeywell (Charlotte, NC, USA). Additionally, acetic acid (p.a) and sodium carbonate (p.a.) were obtained from Honeywell (Charlotte, NC, USA). The C18 powder (40–63 μm, Macherey-Nagel Polygoprep 60-50) was purchased from Fisher Scientific (Waltham, MA, USA) and 1,4-Dioxane from Merck Millipore (Burlington, MA, USA).

### 2.2. Seaweed Samples, Extraction, and Fractionation Procedures

The brown seaweeds *D. dichotoma* and *D. fasciola* were collected in March 2021 when they were most abundant and present in the same location: the Zadar archipelago (Croatia) in the Adriatic Sea. After collection, the samples were washed, freeze-dried using CoolSafe lyophilizer (55-9 PRO model, Labogene, Lillerød, Denmark), and stored in a cool and dark place until analysis. 

Each macroalgae sample was weighed (2 g) and mixed with a methanol (MeOH)/dichloromethane (DCM) (1:1, *v/v*) solvent combination. The extraction was performed in an ultrasonic bath (Bandelin, Sonorex digiplus 560W, Berlin, Germany) for 15 min, repeated three times, and centrifuged between sonication. All supernatants were collected, filtered, and mixed with a small amount of C18 powder. The obtained extracts were subjected to evaporation under nitrogen (5.0, Messer, Croatia) flow to remove the organic solvent. Dry extracts were placed on the preconditioned (methanol and water) SPE cartridge (C18, Agilent Bond Elut, Germany) and the fractionation with solvents of different polarity was performed (water > water: methanol > methanol > dichloromethane). Less polar F3 (methanol) and F4 (dichloromethane) fractions were collected, dried, and stored until further analysis. Polar F1 (water) and F2 (dH_2_O: methanol) fractions were not further analyzed. After drying, the fractions were dissolved in appropriate solvents: F3 in methanol and the F4 fraction in DMSO for biological analysis, or dichloromethane for chromatographic analysis. The obtained fractions F3 and F4 were further used in non-target screening and individual pigment analysis by liquid chromatography and also tested for their antioxidant, antimicrobial, enzymatic activity, and toxicity.

The extraction procedures for determining protein, carbohydrate, total pigment content, and fatty acid analysis are described in each method description section later in the text.

### 2.3. Total Protein and Carbohydrate Content Determination

Total protein content was determined by the Lowry method [23], with slight modifications. Briefly, 200 mg of freeze-dried macroalgae sample was ultrasonicated in alkalic water for 1 h at 40 °C, after which the sample was centrifuged at 4200 rpm/10 min. Pellets were discarded and the supernatant was collected. After cooling, the Lowry mixture was added, mixed, and incubated for 15 min at room temperature. The 1.0 N phenol reagent was added and after 30 min, absorbance was measured (750 nm). Bovine serum albumin (BSA) was used as standard and for possible interference of the sample matrix. 

Total carbohydrate content was determined by a phenol–sulfuric acid method [24]. A reaction was performed by mixing the sample, concentrated sulfuric acid (rapidly), and 5% phenol. Before the absorbance measurement at 490 nm, the samples were incubated for 5 min at 90 °C and then cooled to room temperature. D-glucose was used as the standard and the results were expressed as mg/g.

### 2.4. Identification and Quantification of Algal Pigments Using Spectrophotometry 

Chlorophylls a and b, carotenoids, and pheophytins a and b were determined using the previously described method [25] with slight modifications. The freeze-dried algal sample was ground into powder and 2 mL of methanol was added. Samples were sonicated in an ultrasonic bath for 5 min and centrifuged at 10,000 rpm for 5 min. The procedure was repeated until the biomass (pellet) was colorless, and the chlorophyll and carotenoid content were determined from supernatants using the equations provided by Lichtenthaler [25].

### 2.5. Amino Acid Determination by Ultra-High-Performance Liquid Chromatography (UHPLC)

#### 2.5.1. Extraction of Total Amino Acids (Acid Hydrolysis)

To obtain the amino acid composition and concentrations in samples, acid hydrolysis was performed following the method described by Machado et al. [26]. The hydrolysis of algae samples was performed in closed, de-aerated tubes in a thermoblock (Eppendorf ThermoMixer C, Hamburg, Germany) at 100 °C for 24 h. Afterward, the supernatant was neutralized with borate buffer (0.4 M, pH 10.2), and internal standards norvaline and sarcosine (5 nmol/µL) were added. 

#### 2.5.2. UHPLC Analysis of Amino Acids 

The amino acids were determined using an Agilent UHPLC 1290 Infinity II (Agilent, Santa Clara, CA, USA) instrument equipped with a diode array detector (DAD). The pre-column automatic derivatization of amino acids was performed online in the autosampler needle using the 1290 Multisampler according to the method described in Amino Acid Analysis: “How-To” Guide [27]. The primary amino acids were derivatized with OPA reagent and monitored at 338 nm, while secondary amino acids (hydroxyproline, sarcosine, and proline) were derivatized with FMOC and monitored at 262 nm. For the separation, an AdvanceBio AAA LC analytical column (4.6 × 100 mm, 2.7 µm, Agilent, USA) connected with an AdvanceBio AAA guard column (4.6 × 5 mm, 2.7 µm, Agilent, USA) was used and kept at a constant temperature of 40 °C with an injection volume of 1 µL. The two chromatography mobile phases involved were A (10 mM Na_2_HPO_4_ and 10 mM Na_2_B_4_O_7_ × 10H_2_O, pH = 8.2) and B (acetonitrile:methanol: water, 45:45:10, *v/v/v*) with a flow rate of 1.2 mL/min. The gradient elution program was as follows: 0–0.35 min, 2% B, increasing to 57% B until 13.4 min and continuing to 100% B solution in 13.5 min, followed by 100% B until 15.7 min, and decreasing at 15.8 min to 2% B, then maintained at 2% B for 2 more minutes. OpenLab CDS software was used for instrument control, data acquisition, and data analysis (integration, peak areas, and retention times).

### 2.6. Fatty Acid Determination by Gas Chromatography (GC)

#### 2.6.1. Extraction of Total Lipids and Lipid Transmethylation 

Total lipid extraction from *D. dichotoma* and *D. fasciola* was performed according to Kumari et al. [28] with slight modifications. Briefly, 50 mg of lyophilized macroalgae sample was mixed with 3 mL chloroform:methanol:50 mM phosphate buffer (1:2:0.8, *v/v/v*) solvent mixture, vortexed, and centrifugated for 15 min. The supernatants were collected and extraction of residues was repeated with a solvent mixture chloroform:methanol:50 mM phosphate buffer (1:1:0.8, *v/v/v*). The supernatants were combined, filtered through a 0.45 µm filter, and washed with 2 mL of 50 mM phosphate buffer. After centrifugation, a lower organic layer containing lipids was collected and evaporated to dryness under nitrogen flow. 

To obtain fatty acid methyl esters (FAMEs), the transmethylation of lipid samples was performed. In the dry sample, 1 mL of 1% NaOH in MeOH and tridecanoic acid methyl ester (C13:0ME) as an internal standard for the transmethylation procedure were added and the mixture was incubated for 15 min at 55 °C. Then, 2 mL of 5% methanolic HCl was added and incubated again. Finally, 1 mL Milli-Q water was added, and FAMEs were extracted by adding hexane (3 times/1 mL). The organic layer was dried under nitrogen flow and resuspended in 200µL of hexane with the addition of 5 µL of analytical internal standard pentadecane.

#### 2.6.2. GC-FID Analysis of Fatty Acids

The fatty acids methyl esters (FAMEs) were determined using an Agilent 8890 gas chromatograph (Agilent; Santa Clara, CA, USA) equipped with a fused silica capillary column (J&W DB-WAX Ultra Inert, 60 m × 0.320 mm × 0.50 μm; Agilent, Santa Clara, CA, USA) and flame ionization detector (FID). The sample volume of 1 μL was injected with a split ratio of 1:2.5. The carrier gas was helium. The injector and detector temperatures were 250 and 260 °C, respectively. The initial oven temperature was programmed at 80 °C/1 min, raised to 175 °C at a rate of 25 °C/min, then from 175 °C to 185 °C at a rate of 10 °C/min with hold for 5 min, and then up to 235 °C at a rate of 15 °C/min, with a hold time of 5 min. In the end, the temperature was raised to 250 °C at a rate of 15 °C/min and the hold time was 27 min. OpenLab ChemStation was used for instrument control, data acquisition, and data analysis (integration, peak areas, and retention times). Identification of FAME was obtained by internal calibration using the commercially available FAME Standard Calibration Mix C8:0–C240. 

### 2.7. Non-Targeted Screening by High-Performance Liquid Chromatography–High-Resolution Mass Spectrometry (HPLC-ESI-HRMS) 

HPLC-HRMS analysis of the fractions F3 and F4 from two Dictyota species was performed using an HPLC system LC-30 Nexera (Shimadzu, Kyoto, Japan) equipped with an SPD-M20A diode array UV-VIS spectrophotometric detector and combined with a quadrupole-time-of-flight (QTOF) mass spectrometer TripleTOF 5600+ (AB Sciex, Concord, ON, Canada) with Duospray ion source. Chromatographic separation was achieved using a Nucleodur PFP column (Macherey-Nagel, Düren, Germany), 150 × 2 mm, 1.8 µm, packed with pentafluorophenyl stationary phase, in a gradient elution mode. The mobile phase composition included deionized high-purity Milli-Q H2O (with 0.1% formic acid) and acetonitrile (with 0.1% formic acid), while the gradient program was as follows: 0–3 min 10% acetonitrile, 3–40 min increase in acetonitrile content up to 100%, 40–45 min 100% acetonitrile. The flow rate was 0.3 mL/min, the column temperature was 40 °C, and the injection volume was 5 µL. Ion source parameters included electrospray ionization in positive and negative ion modes (ESI+ and ESI-), curtain gas (CUR) pressure of 30 psi, nebulizing and drying gas pressure (GS1 and GS2) of 40 psi, temperature of 300 °C, voltage (ISVF) of 5500 V (−4500 V in negative ion mode), and a declustering potential (DP) of 80 V. Mass scale was calibrated immediately before each chromatographic run using sodium formate standard solution in an *m/z* range of 100–2000 which provided a mass accuracy better than 5 ppm (10 ppm in MS/MS spectra in the low-mass region). 

The non-targeted screening was performed in the Information-Dependent Acquisition (IDA) mode. The mass range in TOF-MS mode (MS1) was from 100 to 1000 Da. Precursor ions with signal intensity greater than 100 cps were fragmented automatically. Collision-induced dissociation (CID) was performed using collision energy (CE) of 40 eV with a CE spread of 20 eV. Mass range in product ion mode (MS/MS): 20–1000 Da. The instrument control and data acquisition were carried out using Analyst 1.8 TF software (ABSciex, Concord, ON, Canada). The further treatment of the obtained data with the search and tentative identification of the detected compounds was performed using PeakView and FormulaFinder software packages (ABSciex, Concord, ON, Canada). To determine the elemental compositions of analytes, their exact masses and isotopic distributions were used with the following constraints on the number of atoms: C—1–100, O—0–50, H—0–200, N—0–5, P—0–2. The search for potential candidates was carried out in ChemSpider, Pub-chem, Lotus, and Lipid Maps online databases followed by ranking according to the degree of conformity of the proposed structure to the obtained tandem mass spectrum. In some cases, manual data processing and the tentative identification of the analytes based on the known patterns of CID fragmentation were used.

### 2.8. Pigment Determination in F3 and F4 Fractions by High-Performance Liquid Chromatography (HPLC)

The obtained fractions F3 and F4 from *D. dichotoma* and *D. fasciola* were diluted and filtered through a 0.22 µm PTFE syringe filter before HPLC analysis. Samples were analyzed on an HPLC Agilent 1100 Series System (Agilent, USA) instrument equipped with a variable wavelength detector (VWD). A Thermo Scientific Acclaim C30 reverse-phase analytical column (25 cm, 4.6 mm i.d., 5 µm) was used for the separation at 20 °C. HPLC analysis was performed according to Cikoš et al. [2] but with slight modifications. Briefly, separation was performed with mixture (A) methanol (MeOH), isopropanol, and Mili-Q water (80:17:3, *v/v/v*) and mixture (B) MeOH and isopropanol (20:80, *v/v*) following gradient elution: 0 min, 0% B, 20 min, 100% B, 30 min 100% B, 35 min, 0% B, while the flow rate was as follows: 0–20 min, 0.9 mL/min, 20–30 min, 0.9–1 mL/min, and 30–35 min, 1 mL/min. The detection wavelength was 450 nm, the injection volume was 5 μL, and the total runtime was 35 min. The pigments were identified by comparing the retention times of separated peaks with those of standards. Calibration curves for seven standards (fucoxanthin, astaxanthin, lutein, canthaxanthin, β-carotene, chlorophyll a, and chlorophyll b) were prepared and used for quantification in samples (external standard method). Stock solutions of standards were prepared by dissolving in acetone. Working standards were prepared in the 6.25–500 µg/mL range. The limits of detection (LOD) and quantification (LOQ) were calculated from the parameters obtained from the calibration curve using the equations LOD = 3 s/b and LOQ = 10 s/b, where *s* is the standard deviation of the y-intercept of the regression line and *b* is the slope of the calibration curve [29]. The results were calculated as mean values ± standard deviation (SD) and expressed as mg/g of the dry fraction.

### 2.9. Zebrafish Embryotoxicity Test (ZET)

Zebrafish *Danio rerio* adults (wildtype WIK strain obtained from the European Zebrafish Resource Center of the Karlsruhe Institute of Technology, Germany) were maintained and spawned as specified by Babić et al. [30]. The toxic potential of tested macroalgal fractions was determined by performing a zebrafish embryotoxicity test [31]. Dose-range-finding experiments were conducted on a wide range of concentrations prepared in serial dilutions (0.1865–0.005 mg/mL of F3 fractions, 0.01–0.0003 mg/mL of F4 fractions) using 10 embryos per tested concentration in 3 replicates. Exposed specimens were incubated under constant conditions (regulated light/dark photoperiod (14/10) and temperature of 26 °C (Innova 42 incubator shaker, New Brunswick, NB, Canada)) [30]. The survival and developmental abnormalities were recorded at 96 h post-fertilization using an Olympus CKX41 inverted microscope equipped with LAS EZ 3.2.0 software and a digital camera Leica EC3.

### 2.10. Antioxidant Activity Assays

#### Antioxidant Activity Determination In Vitro

Stock solution and proper dilutions of F3 and F4 fractions were made and used in all methods. The used methods were 2,2-diphenyl-1-picrylhydrazyl-hydrate (DPPH) assay, reduction of radical cation assay (ABTS), oxygen radical absorbance capacity (ORAC) assay, and ferric-reducing antioxidant power (FRAP) assay. Using a microplate reader (Spectramax ABS plus, Molecular Devices, San Jose, CA, USA), spectrophotometric measurements were performed in triplicate for all mentioned assays. The antioxidant activity of F3 and F4 fractions was tested by the reactions with appropriate reagents. The change of color regarding the control or blank sample after incubation was measured and the results were expressed as mean ± standard deviation (*n* = 3). A detailed methodology of the used assays is described in a recent publication of our research group [32].

### 2.11. Antimicrobial Activity

The broth microdilution test was used to assess the antimicrobial activities of algal fractions according to the Clinical & Laboratory Standards Institute guidelines [33]. Stock fractions were diluted to 1% of solvent (MetOH or DMSO). Tested bacterial species were *Staphylococcus aureus* ATCC 6538 and *Escherichia coli* NCTC 12241. Positive (inoculated media without the tested sample with 1% of solvent) and negative (sterile media with 1% of solvent) controls were used for all tests, as well as quality control with chloramphenicol. Optical density was measured at 600 nm after overnight growth at 35 °C. The obtained values were normalized to positive control growth. 

### 2.12. Enzymatic Activity

#### 2.12.1. Anti-Collagenase Activity

In vitro collagenase inhibition assay was performed following the manufacturer’s instructions. Each fraction was tested in 5 serial dilutions (2–0.15 mg/mL). 1,10-phenanthroline was used as a positive inhibitor. The rate of enzymatic hydrolysis of the FALGPA substrate was monitored in terms of absorbance change at 345 nm (Infinite 200 PRO spectrophotofluorimeter, Tecan, Austria). Reactions were performed in triplicate.

#### 2.12.2. Antityrosinase Activity

Inhibition of tyrosinase (monophenolase) and L-DOPA auto-oxidation (diphenolase) was evaluated based on the previously described procedure [34]. Briefly, the extracts were dissolved in a suitable solvent (methanol for F3 and DMSO for F4 fraction, respectively) in the highest possible concentration, while the working solution was made up to 1% solvent using 50 mM PBS (pH 6.8). Kojic acid was used as a positive control, while the substrates were 2 mM L-tyrosine and 0.5 mM L-DOPA for the determination of monophenolase and diphenolase activity, respectively. The reaction was followed in kinetic mode for 30 min, in a 96-well plate where 70 μL of each diluted sample concentration (in triplicate) and 30 μL of tyrosinase (333 Units/mL in phosphate buffer) were added. After incubation at room temperature for 5 min, 110 μL of the substrate was added to each well. The optical density was monitored at 475 nm. Afterward, the percentage of inhibition was calculated.

#### 2.12.3. α-Amylase Inhibitory Activity 

The anti-diabetic properties of macroalgal fractions were determined by the Caraway–Somogyi iodine/potassium iodide (IKI) method with minor modifications [35,36]. Stock solutions of macroalgal fractions and acarbose (positive control), as well as α-amylase solution (0.5 mg/mL) and starch solution (0.05%), were prepared in phosphate buffer (10 mM; pH 6.9 with 0.006 M sodium chloride). Sample blanks and negative control were also prepared. In a 96-well plate, 25 μL of the sample or acarbose and 50 μL of 0.05% starch solution were mixed. Afterward, 50 μL of α-amylase solution (0.5 mg/mL) was added and the plate was incubated for 15 min at 37 °C. The reaction was stopped by adding 25 μL of HCl (1 M), followed by the addition of 100 μL iodine–potassium iodide solution. All reactions were performed in triplicate. The absorbance was read at 630 nm and the α-amylase inhibitory activity (%) was calculated.

#### 2.12.4. Pancreatic Lipase Inhibitory Activity 

The inhibition of pancreatic lipase was quantified by a colorimetric assay as described by Bustanji et al. [37] with slight modifications. Porcine pancreatic lipase (crude) type II was firstly suspended in tris-HCl buffer (2.5 mmol, pH 7.4 with 2.5 mmol NaCl). Pancreatic lipase was preincubated with each extract for 10 min at 37 °C and the reaction started by adding the substrate to the reaction mixture. The substrate, p-nitrophenyl palmitate (PNPP), was dissolved in Tris-Na deoxycholate buffer (50 mM Tris-HCl, pH 8 and 5 mM Na-deoxycholate). The activity of pancreatic lipase was defined as an increase in the rate of p-nitrophenol release. The increase may be estimated from the slope of the linear segment of (absorbance vs. time) profiles. The percentage of residual activity was determined for each fraction by comparing the lipase activity of pancreatic lipase with and without the extract. The inhibitor of pancreatic lipase, orlistat, was used as a positive control in the assay mixture. The final concentration of solvent was fixed and did not exceed 5%.

#### 2.12.5. Acetylcholinesterase Inhibitory Activity

The F3 and F4 fractions of *D. dichotoma* and *D. fasciola* were tested for their acetylcholinesterase (AChE) activity with the Ellman method [38]. Briefly, the reaction mixture consisted of 100 μL 3 mM DTNB (Ellman’s reagent), 20 μL AChE (0.26 U/mL), 40 μL 50 mM Tris buffer, pH 8, and 20 μL algal fractions at various dilutions of stock solution (27.5 mg/mL). The 96-well plate was incubated at room temperature for 15 min when the absorbance was measured at 412 nm. The AChE inhibitor tacrine was used as a control for inhibitory activity at a final concentration of 100 nM. The enzymatic reaction was initiated by the addition of 20 μL 15 mM ATCI (acetylcholine iodide) and hydrolysis of acetylthiocholine was monitored at 5 min intervals for 25 min. All reactions were performed in triplicate. The percentage of inhibition at the end of the reaction was calculated.

### 2.13. Statistical Analysis

All obtained values for the evaluation of antimicrobial and antioxidant activities are expressed as mean values with standard deviations of three replicates. Using GraphPad Prism 8.0 (GraphPad Software Inc., San Diego, CA, USA), the differences between the means were analyzed by Sidak’s multiple comparisons test of Two-Way ANOVA for amino acid and fatty acid results, while Tukey’s test of One-Way ANOVA was used for all other obtained results. Significantly different values were considered those of *p* < 0.05 and lower. Additionally, using the regression program in GraphPad Prism 8.0, correlations and regression analysis between spectrophotometric methods ABTS, ORAC, DPPH, and FRAP were performed.

## 3. Results

### 3.1. Phytochemical Content of D. dichotoma and D. fasciola (Total Protein, Carbohydrate, and Chlorophyll Content)

A phytochemical characterization regarding total protein, carbohydrate, and chlorophyll content is shown in Table 1. Around two-fold higher protein content was determined by the Lowry method in *D. dichotoma* when compared to the *D. fasciola* sample. 

Additionally, somewhat higher carbohydrate content was obtained for the *D. dichotoma* than for the *D. fasciola* sample. The results for chlorophylls and carotenoids are also shown in Table 1. Chlorophyll a was the dominant pigment in both algae samples, but it was almost three-fold higher for *D. dichotoma*. Chlorophyll derivatives, pheophytin a, and pheophytin b were present in both samples with higher content of pheophytin a. However, an almost seven-fold higher concentration was obtained in the *D. dichotoma* sample.

### 3.2. Amino Acid Profile Determined by Ultra-High-Performance Liquid Chromatography (UHPLC)

**Figure 1:**
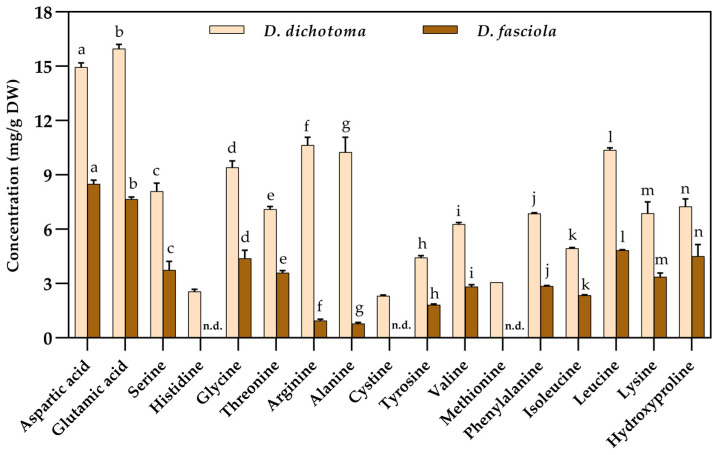
The amino acid composition of *D. dichotoma* and *D. fasciola* determined by UHPLC analysis. Common letters indicate a significant difference between the samples (*p* < 0.0001). n.d. = not detected.

All 21 primary and secondary amino acids had a linear response range from 45 to 900 pmol/µL with an average correlation coefficient (r) of 0.995 ± 0.016. The accuracy of the developed method was 2.11%. The minimal and maximal limits of detection were 0.014 and 0.267 µg/mL, while the minimal and maximal limits of quantification were 0.042 and 0.809 µg/mL, respectively. The amino acid composition of *D. dichotoma* and *D. fasciola* is presented in Figure 1. There were 17 amino acids identified in the *D. dichotoma* and 15 amino acids in the *D. fasciola* sample after UHPLC analysis. The most abundant amino acids in the *D. dichotoma* sample were glutamic (15.96 ± 0.24 mg/g DW) and aspartic (14.96 ± 0.24 mg/g DW) acids, while almost the same amount of arginine (10.65 ± 0.42 mg/g DW), leucine (10.37 ± 0.12 mg/g DW), alanine (10.24 ± 0.83 mg/g DW) and glycine (9.40 ± 0.35 mg/g DW) was observed. The amount of other amino acids was two-fold lower. The results for *D. fasciola* revealed that the most abundant amino acids were also aspartic (8.49 ± 0.21 mg/g DW) and glutamic (7.66 ± 0.11 mg/g DW) acids, but with almost two-fold lower concentration (*p* < 0.0001) when compared to *D. dichotoma*. Leucine (4.84 ± 0.03 mg/g DW), hydroxyproline (4.50 ± 0.65 mg/g DW), and glycine (4.38 ± 0.46 mg/g DW) were the next most abundant, but also with two-fold lower concentration (*p* < 0.0001) than in *D. dichotoma*. There was a statistically significant difference (*p* < 0.0001) between the amounts of all amino acids present in *D. dichotoma* and *D. fasciola* samples, with higher concentrations found for *D. dichotoma* (Figure 1). 

The biggest difference among samples was observed for alanine and arginine; namely, 13-fold and 11.1-fold higher amounts were found in *D. dichotoma* than in *D. fasciola*. Additionally, in contrast to the *D. dichotoma* sample, histidine, cystine, and methionine were not determined in the *D. fasciola* sample. Nine of ten essential amino acids were found in *D. dichotoma* (58.7 ± 1.63 mg/g DW), corresponding to around 44% of the total amount (131.32 ± 4.25 mg/g DW). In comparison, seven essential amino acids were found in *D. fasciola* (20.79 ± 0.62 mg/g DW), corresponding to 40% of the total amount (52.18 ± 2.62 mg/g DW). 

### 3.3. Fatty Acid Profile Determined by Gas Chromatography (GC) 

A linear response for all 37 fatty acids (standards) was obtained in the range of 0.7–10 mg/mL with an average correlation coefficient (*r*) of 0.992 ± 0.003. The accuracy of the developed GC method was 4.83%, while minimal and maximal limits of detection were 0.111 and 0.452 µg/mL, respectively. The minimal and maximal limits of quantification were 0.336 and 0.923 µg/mL, respectively. The fatty acid composition of *D. dichotoma* and *D. fasciola* algae is presented in Table 2. In the *D. dichotoma* sample, 21 fatty acids were identified after GC-FID analysis, while 15 fatty acids were found in the *D. fasciola* sample. 

In both samples, saturated fatty acids (SFA) were the most represented, with values of 38.99 ± 0.45% for *D. dichotoma* and 45.90 ± 3.90% for *D. fasciola*. Additionally, nearly the same amount of monounsaturated fatty acids (MUFA) was found in both macroalgae: 28.95 ± 1.67% and 28.17 ± 1.18% for *D. dichotoma* and *D. fasciola*, respectively. In the *D. dichotoma* sample, higher content (29.57 ± 0.43%) of polyunsaturated fatty acids (PUFA) was found when compared to *D. fasciola* (27.23 ± 3.03).

The main fatty acids present In *D. dichotoma* were myristic, dihomo-γ-linolenic acid, and palmitoleic acid, with mean values of 16.68 ± 0.23%, 14.92 ± 0.13%, and 11.65 ± 0.13%, respectively. In contrast, the main fatty acids determined in *D. fasciola* were palmitic acid, oleic acid, and docosadienoic acid, with mean values of 27.49 ± 1.72%, 24.37 ± 0.76%, and 10.6 ± 1.01%, respectively. Seven fatty acids, namely myristoleic acid, α-linolenic acid, linolelaidic acid, lauric acid, eicosadienoic acid, heneicosylic acid, and arachidonic acid, were not detected in *D. fasciola*. At the same time, they were present in the *D. dichotoma* sample in a percentage of 1.25 and 4.50%. The only distinguishing factor between the two samples was the existence of docosadienoic acid (C22:2 (c13, c16)), which is a well-known omega-6 fatty acid found in the *D. fasciola* sample at a concentration of 10.6 ± 1.01%, whereas it was not identified in the *D. dichotoma* sample.

### 3.4. Non-Targeted Screening of Less Polar Compounds

The chemical composition of the lipophilic fractions of brown algae fractions at the molecular level is extremely complex and cannot be fully established. However, the non-targeted screening strategy using the HPLC-HRMS technique with information-dependent MS/MS data acquisition provides valuable information on key components, complementing the results of the targeted determination of priority constituents described in the previous sections. Analysis of the obtained chromatograms of the fractions F3 and F4 made it possible to detect hundreds of individual compounds, 48 of which, with the most intense signals on the chromatograms, were tentatively identified or assigned to a certain class based on their elemental compositions and tandem mass spectra (Table 3).

The F3 fraction predominantly contained more polar compounds with shorter retention time (RT) on the reversed stationary phase (Table 3). On the contrary, the F4 fraction was characterized by the dominance of the most lipophilic components, which had a retention time of more than 30 min. As expected, the detected major components mainly belonged to the three classes of natural compounds: lipids, diterpenoids, and chlorophyll derivatives, while another major class of algal pigments—carotenoids—was represented only by fucoxanthin.

Along with mono-, di-, and triacylglycerides, which gave relatively weak signals on the obtained chromatograms and thus were not included in Table 3 (an exception is glyceryl stearate), several major compounds belonging to glycosylacylglycerols, the sugar moiety which is represented by galactose or its dimer [39], were found. These include digalactosylmonoacylglycerols (DGMG), monogalactosylmonoacylglycerols (MGMG), digalactosyldiacylglycerols (DGDG), and monogalactosyldiacylglycerols (MGDG). Some of them show two peaks in the extracted ion chromatograms, indicating the presence of positional isomers. A specific feature of the detected glycolipids is the presence in the mass spectra of both signals of protonated ([M + H]^+^) and cationized with ammonium ion ([M + NH_4_]^+^) molecules. The latter, in most cases, were the dominant ions. The most polar representatives of glycolipids (DGMG and MGMG) were found exclusively in the F3 fraction and predominated in extracts of *D. dichotoma*, while the less polar representatives of MGDG in the F4 fraction were found in the highest amounts in *D. fasciola*. Fatty acids in the detected glycolipids are presented by stearidonic (18:4), oleic (18:1), palmitoleic (16:1), palmitic (16:0), and myristic (14:0) acids, which is evidenced by the loss of corresponding neutral molecules observed in tandem mass spectra. Of the free fatty acids, only oleic acid was detected in the F3 fraction. 

Oleamide and erucamide were also found in significant amounts. In addition to glycolipids, the fraction F4 also contained two structurally similar representatives of neutral sphingolipids (compounds No. 36 and 37 with elemental compositions of C_44_H_83_NO_9_ and C_46_H_85_NO_9_, respectively), which predominated in the *D. fasciola* extract.

The diterpenoids are characterized by similar tandem mass spectra, in which the primary loss of water and the elimination of other functional groups, followed by the destruction of the carbon skeleton with the formation of peaks of hydrocarbons separated by 14.0157 Da (methylene group) are observed. Establishing the structure of these compounds is highly challenging and unreliable if analytical standards are not accessible. Nevertheless, the information on the number of hydroxyl, carbonyl, carboxyl, and acetyl groups obtained in MS/MS experiments in most cases allows for attributing them to certain specific compounds based on the available literature data. It should be noted that there is a fundamental difference in the composition of major diterpenoids in the extracts of the two studied algae. The fraction F3 of *D. dichotoma* extract is characterized by the presence of the two major compounds not found in *D. fasciola* and identified as the dolastane diterpenoids dichotenone and amijitrienol. In turn, *D. fasciola* is distinguished by the presence of a set of similar compounds (No. 17, 18, 20) in the F3 fraction, which accounts for about 45% of the total area of the chromatographic peaks of all compounds presented in Table 3. They were tentatively identified as derivatives of diterpene dolabelladiene containing hydroxy groups, some of which were acetylated. 

Among the less polar compounds of the F4 fraction, several steroids (No. 21, 24, 29) were also found, which, like dolabelladienes, predominated in the *D. fasciola* extract. Being the least polar analytes, six chlorophyll-related compounds, pheophytin derivatives (No. 43–48) with the elemental composition C_55_H_74(72)_N_4_O_5-9_, were the last to be eluted from the chromatographic column and, accordingly, sharply predominated in the F4 fraction. Pheophorbide A and its hydroxylated derivative (No. 34 and 31, respectively) were also found in small amounts. They are characterized by a significantly shorter retention time due to the absence of a long hydrocarbon chain in their structure. 

Among the detected compounds that are not included in the mentioned large groups, loliolide should be noted. The carotenoid catabolites formed due to fucoxanthin degradation, the monoterpene lactone loliolide, and its positional isomer have often been found in brown algae extracts [40,41].

### 3.5. Pigment Determination in F3 and F4 Fractions

HPLC analysis of the pigments was performed on fractions F3 and F4 of *D. dichotoma* and *D. fasciola* with the solvents methanol and dichloromethane after the removal of water-soluble components that were eluted in the fractions 1 (F1; H_2_O) and 2 (F2; MeOH: H_2_O, 1:1, *v/v*). Before the sample analysis, a mixture of seven standards (fucoxanthin, astaxanthin, lutein, canthaxanthin, β-carotene, chlorophyll a, and chlorophyll b) was analyzed to obtain calibration curves. The linear response was obtained for all standards in the 6.25–500 µg/mL range with the correlation coefficients (*r*) of 0.999. The detection limits for the three pigments found in our samples, namely fucoxanthin, lutein, and chlorophyll a, were 2.28, 1.59, and 4.84 µg/mL, while the limits of quantification were 6.92, 4.81, and 14.68 µg/mL, respectively. 

As can be seen in Table 4, the major pigments—fucoxanthin, lutein, and chlorophyll a—were found in the F3 and F4 fractions of two Dictyota species. Their detection was dependent on the applied solvent. Therefore, fucoxanthin was the main detected peak in the F3 fraction of both *D. dichotoma* and *D. fasciola* in the amount of 5.01 ± 0.63 mg/g and 3.15 ± 0.96 mg/g, respectively, while a small amount of lutein was found only in F3 of *D. fasciola*, with the concentration of 0.27 ± 0.06 mg/g. In the less polar fraction F4, the main pigment identified was chlorophyll a in both Dictyota species, but the concentration was almost three-fold higher (*p* < 0.05) in F4 of *D. dichotoma* (1.31 ± 0.21 mg/g) than in *D. fasciola* (0.44 ± 0.04 mg/g), while a small amount of fucoxanthin (0.35 ± 0.01 mg/g) in the F4 fraction of *D. dichotoma* was also detected.

### 3.6. Zebrafish Embryotoxicity Test (ZET)

The F4 fractions of both tested macroalgae demonstrated no negative effects on embryonic development within the tested concentration range (0.01–0.0003 mg/mL of *D. fasciola* and 0.1865–0.005 mg/mL of *D. dichotoma* fractions). On the contrary, F3 fractions decreased survival and induced developmental alterations. 

**Figure 2:**
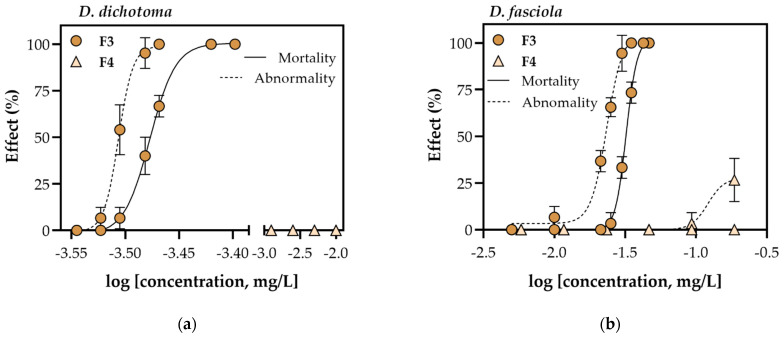
Dose–response curves showing cumulative mortality and developmental abnormality of zebrafish *D. rerio* at 96 h of exposure to two fractions (F3 and F4) of (**b**) *D. fasciola* and (**a**) *D. dichotoma*. Error bars indicate standard deviations (SD).

*D. dichotoma* F3 fraction demonstrated the highest toxicity (50% lethal concentration (LC_50_) = 0.333 × 10^−3^ mg/mL, and 50% effective concentration (EC_50_) = 0.3116 × 10^−3^ mg/mL; Figure 2a,b; Table 5). Morphological abnormalities observed on the *D. fasciola* F3 fraction mainly included pericardial edema and yolk sac edema, while the *D. dichotoma* F3 fraction mainly caused scoliosis. No significant mortalities and abnormalities (<5%) were observed in control groups (artificial water and solvent controls (0.1% of DMSO and MeOH)).

### 3.7. Antioxidant Activity Determination “In Vitro”

**Figure 3:**
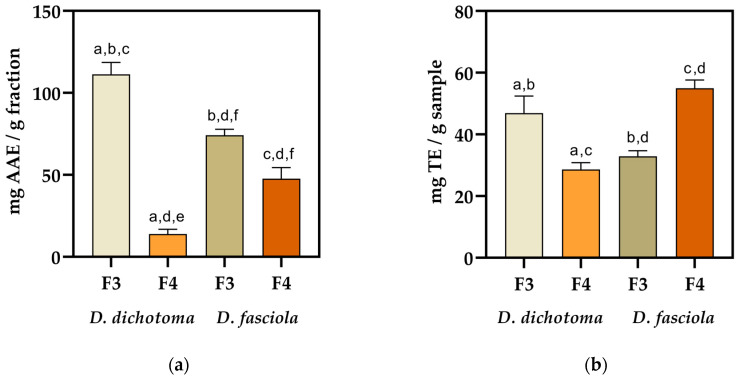

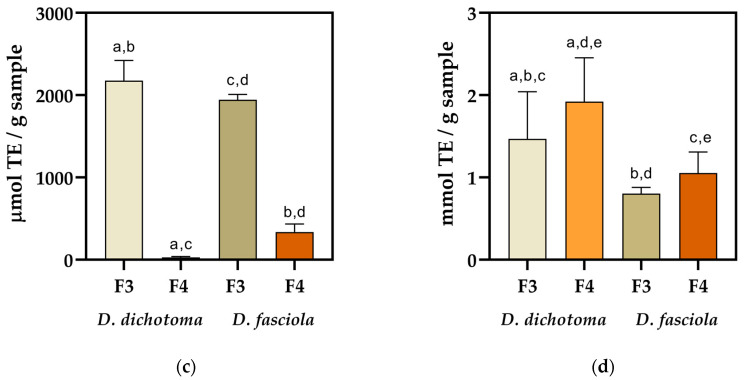
Radical scavenging effect of *D. dichotoma* and *D. fasciola* F3 and F4 fractions using (**a**) 2,2-diphenyl-1-picryl-hydrazyl-hydrate (DPPH), (**b**) reduction of radical cation (ABTS), (**c**) oxygen radical absorbance capacity (ORAC), and (**d**) ferric reducing antioxidant power (FRAP) in vitro assays (mean ± SD; *n* = 3). Common letters indicate a significant difference between the fractions (*p* < 0.05).

Antioxidant activity of the different polarity F3 and F4 fractions from two brown macroalgae varied according to the used method. Four spectrophotometric methods were performed: DPPH, ABTS, ORAC, and FRAP (Figure 3a–d). 

By implementing the DPPH assay, the F3 fraction of both macroalgae showed moderate antioxidant activity with inhibition around 35% for *D. dichotoma* (5 mg/mL) and 22% for *D. fasciola* (5 mg/mL). DPPH assay results revealed that the *D. dichotoma* F3 fraction showed the highest antioxidant activity when normalized per gram of the fraction (109.1 ± 9.2 mg/g AAE), followed by *D. fasciola* F3 (73.0 ± 4.4 mg/g AAE) and *D. fasciola* F4 (45.7 ± 7.3 mg/g AAE) fractions (Figure 3a). Similar antioxidant activity was obtained for the *D. fasciola* F4 (54.9 ± 2.7 mg/g TE) and *D. dichotoma* F3 (46.9 ± 5.5 mg/g TE) fractions by implementing an ABTS assay, while the lowest activity was obtained for the *D. dichotoma* F4 fraction (28.7 ± 2.2 mg/g TE) (Figure 3b). Additionally, *D. dichotoma* showed inhibition around 40% (2.5 mg/mL) for the F3 fraction and around 28% for F4 at the same concentration. *D. fasciola* F3 fraction showed inhibition of around 24% (2.5 mg/mL), while the F4 fraction showed the highest inhibition around 43% (2.5 mg/mL). To obtain the concentration at which 50% of inhibition is achieved (IC_50_), different concentrations of fractions were tested ranging from 0.025 to 10 mg/mL. Calculated IC_50_ values, confidence intervals, correlation coefficients (R^2^), and Hillslope values are shown in Table 6. The highest antioxidant activity, i.e., the lowest IC_50_ value was obtained for the *D. fasciola* F4 fraction (2.372 mg/mL) while the high IC_50_ value of the *D. fasciola* F3 fraction (6.167 mg/mL) indicates lower antioxidant activity. Because the upper inhibition plateau for *D. dichotoma* F4 fraction could not be reached, the IC_50_ value was not determined. Measuring the loss of fluorescence intensity by ORAC assay (Figure 3c), the antioxidant activity of the *D. dichotoma* (1944.2 ± 63.9 μM/g TE) and *D. fasciola* (2175 ± 246.7 μM/g TE) F3 fractions was significantly higher than for F4 fractions (337.5 ± 96.4 μM/g TE for *D. dichotoma* and 30 ± 9.6 μM/g TE for *D. fasciola*). The last implemented method, FRAP in vitro assay, revealed approximately two-fold higher activity for both F3 and F4 *D. dichotoma* fractions than those of *D. fasciola* (Figure 3d).

### 3.8. Antimicrobial Activity Determination

The methanolic and DMSO fractions of tested algal species showed dose-dependent inhibitory activity against Gram-negative and -positive bacteria. However, these responses were about 50% inhibition for the F3 fractions of *D. dichotoma* and *D. fasciola* at the highest tested concentration (877 and 521 µg/mg, respectively) for the Gram-negative bacterium *E. coli*. The F4 fraction of *D. dichotoma* and *D. fasciola* showed lower inhibitory activity against this bacterium. In contrast, F3 from both algae achieved minimal inhibitory concentration (MICs) against the Gram-positive bacterium *S. aureus* of 438 µg/mL for *D. dichotoma* and 521 µg/mL for *D. fasciola*. In addition, EC_50_ was determined for *D. dichotoma* and *D. fasciola*, and the obtained values were 122 ± 5.5 µg/mL and 142.3 ± 13 µg/mL, respectively. Finally, the F4 fractions also showed no significant inhibitory effect on *S. aureus*. 

### 3.9. Enzymatic Activity Determination

#### 3.9.1. Dermatological Potential (Anti-Collagenase and Antityrosinase Activities)

**Figure 4:**
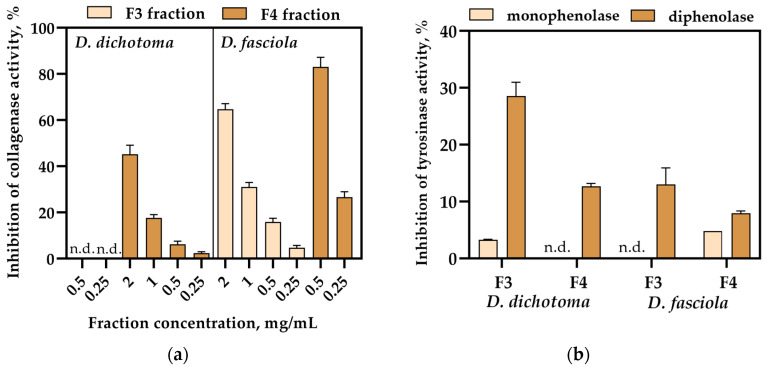
Enzymatic activity of *D. dichotoma* and *D. fasciola* fractions evaluated as (**a**) collagenase inhibitory potential at different concentrations of samples and (**b**) tyrosinase inhibition percentage at the concentration of 0.250 mg/mL for all fractions. Data are expressed as the means ± SD of three independent experiments. n.d. = not detected.

The anti-collagenase activity was detected in all tested fractions except in the *D. dichotoma* F3 fraction (Figure 4a). The highest inhibitory effect was recorded at only 0.50 mg/mL of *D. fasciola* F4 fraction and reached 83.05%. The highest tested concentrations (2 mg/mL) of *D. dichotoma* F4 and *D. fasciola* F3 fractions caused similar but less pronounced effects by inhibiting 45.25% and 64.70% of collagenase activity, respectively.

Tyrosinase inhibitory activity or inhibition of enzymatic browning was evaluated in response to two different substrates, L-tyrosine (monophenolase activity) and L-DOPA (diphenolase activity). Due to the organic solvents (methanol and DMSO) present in fractions F3 and F4, the highest possible tested concentration was 1% of both fractions, so IC_50_ concentration could not be achieved. Algae samples were tested at the concentration of 250 µg/mL and the results are shown in Figure 4b. As can be seen, a rather low inhibition percentage was obtained for both *D. dichotoma* and *D. fasciola*. Similar monophenolase activity was observed for *D. dichotoma* F3 (3.28 ± 0.06%) and *D. fasciola* F4 (4.81 ± 0.01%) fractions, while no activity was observed for *D. dichotoma* F4 and *D. fasciola* F3 fractions. All samples showed better diphenolase activity. The highest activity was observed for *D. dichotoma* F3 (28.56 ± 2.44%), followed by *D. fasciola* F3 (13.00 ± 2.91%) and *D. dichotoma* F4 (12.65 ± 0.54%), while the lowest activity was observed for *D. fasciola* F4 fraction (7.95 ± 0.39%). Positive control was Kojic acid, also tested at the concentration of 250 µg/mL with the inhibition percentage of 100% for monophenolase, and 78.50 ± 1.50% for diphenolase.

#### 3.9.2. Dietary Potential (α-Amylase and Pancreatic Lipase Inhibitory Activity)

**Figure 5:**
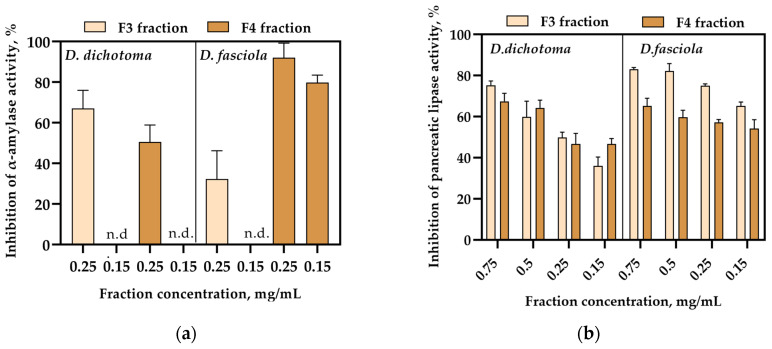
Enzymatic activity of *D. dichotoma* and *D. fasciola* fractions evaluated as (**a**) α-amylase inhibitory activity at the concentrations of 0.25 and 0.15 µg/mL for all fractions and (**b**) pancreatic lipase inhibitory potential at different concentrations of samples. Data are presented as mean ± SD (*n* = 3). n.d. = not determined.

The effects of methanol (F3) and DMSO (F4) *D. dichotoma* and *D. fasciola* fractions on α-amylase inhibitory activity are presented in Figure 5a. As shown, α-amylase inhibitory activity was detected in all samples at a higher tested concentration (0.25 mg/mL). In the case of *D. dichotoma*, the F3 fraction resulted in higher enzyme inhibition (67.05%) compared to the F4 fraction (50.52%). Neither F3 nor F4 fractions exhibited inhibitory activity at a lower tested concentration (0.15 mg/mL). In contrast to *D. dichotoma*, F4 fractions of *D. fasciola* exhibited significantly higher inhibitory activity of 92.03% and 79.75% at both tested concentrations (0.25 mg/mL, and 0.15 mg/mL, respectively) than its F3 fractions. Moreover, the F3 fraction inhibitory activity of 32.27% was detected only at a higher tested concentration (0.25 mg/mL). Acarbose was used as a positive control (100% inhibition for the concentration of 0.25 mg/mL, and 88.31% for the concentration of 0.15 mg/mL.

The pancreatic lipase inhibition percentages of the methanol (F3) and DMSO (F4) extracts are presented in Figure 5b. As can be seen, F3 fractions of *D. fasciola* showed high inhibitory activity on 0.75 mg/mL (83.2%), slightly lower than 1.0 mg/mL (83.8%, data not shown). The high inhibitory activity remained for the five-times diluted concentration (65.3%). The inhibitory potential of *D. fasciola* F4 fraction, compared to the same concentration of F3 fraction (0.15 mg/mL), resulted in 11.1% lower activity (54.2%). Although *D. dichotoma* F4 fractions showed higher inhibitory potential than *D. fasciola* F4 fractions at higher concentrations, the inhibitory potential at lower concentrations of *D. dichotoma* decreased 1.5 times faster than *D. fasciola* fractions. In all tested concentrations, F3 fractions of *D. dichotoma* showed lower inhibitory potential than F3 fractions of *D. fasciola*. Orlistat was used as a positive control (93.2% for concentration 0.15 mg/mL and 100% for higher concentrations).

#### 3.9.3. Neuroprotective Potential (Acetylcholinesterase Inhibitory Activity)

**Figure 6:**
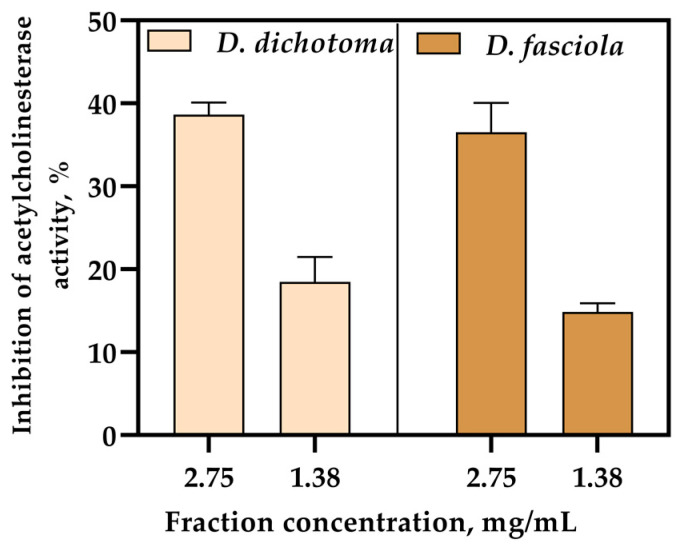
Acetylcholinesterase inhibitory activity of the *D. dichotoma* and *D. fasciola* fractions. Data are expressed as mean ± SD (*n* = 3).

Testing of neuroprotective activity of *D. dichotoma* and *D. fasciola* was only performed on F3 fractions because the DMSO (in which the F4 fractions were re-suspended) itself shows anti-AChE activity [42]. The highest tested concentration for both F3 fractions was 2.75 mg/mL (10% of stock concentration) and similar dose-dependent inhibitory activity was obtained (Figure 6) with 38.64 ± 1.46% for *D. dichotoma* F3 and 36.52 ± 3.52% for *D. fasciola* F3. The AChE inhibitor tacrine showed 100% of AChE under these experimental conditions. 

## 4. Discussion

Various marine organisms, including micro- and macro-algae, are a source of new, safe, and effective agents against different oxidizing substances (i.e., free radicals) [5,18]. Among these, brown algae contain a lot of compounds with high antioxidant activity such as pigments, polyphenols, polysaccharides, proteins, and others [6] which makes them suitable for application in diverse industries (i.e., food, pharmaceutics, etc.). 

Protein levels in macroalgae can vary from 1.7 to 27.6% of dry weight, depending on the harvesting season, location, and nitrogen availability [6]. Barbarino and Lourenco [43] investigated several macroalgae using different methods to determine their total protein content. The authors reported that *D. menstrualis* had a total protein content of 7.01 ± 0.13%, which is consistent with our findings on *D. dichotoma*. Other research reported that the total protein content of *D. caribaea* was 5.6 ± 0.07% [44], which is 1.7-fold higher than our results for *D. fasciola* and 1.3-fold lower than the results obtained on *D. dichotoma* (Table 1). In addition to protein content, carbohydrate content is also very important for many industries, since carbohydrates represent nutritionally beneficial components. It has been reported that marine macroalgae contain 50–60% of carbohydrates [45] and carbohydrate levels in different Dictyota species vary between 10.8 to 54.2% of dry weight [6]. Deyab et al. [46] reported that carbohydrates contributed to 30% of dry *D. dichotoma* biomass, followed by 11.1% of proteins and only 1.3% of total lipids. Additionally, Martins et al. [47] analyzed 24 macroalgae, and carbohydrate content for *D. menstrualis* was around 250 mg/g DW, 3-fold lower than the results obtained for both *D. dichotoma* and *D. fasciola*. The differences between obtained and previously reported results may be attributed to different extraction procedures, location of sample collection, or harvesting season.

Brown macroalgae are known for their high pigment content and nowadays, natural pigments have been used as supplements or additives in several industries [48]. In this research, pigments were firstly determined by the spectrophotometric method for screening their presence in the sample (Table 1). Afterward, they were identified by non-target screening by HPLC-HRMS (Table 3), and finally, some of them were quantified by HPLC (Table 4). Chemical analysis of less polar fractions (F4) of both macroalgae *D. dichtoma* and *D. fasciola* by implementing all three mentioned methods revealed the presence of pheophytin a, a known chlorophyll a derivative. Deyab et al. [46] reported chlorophyll a as the most represented pigment (55% of the total pigment content) in *D. dichotoma*. Herein, chlorophyll a was present in F4 fractions of both algae, and it was 3.25-fold higher for *D. dichotoma* than for the *D. fasciola* F4 sample. This is in agreement with the literature data, where chlorophyll a was determined in *D. bartayresiana* and *D. cervicornis* (5 mg/g DW and 3.8 mg/g DW, respectively) [9]. Furthermore, pheophorbide a, also a chlorophyll a derivative, was detected in both *D. dichotoma* and *D. fasciola* F4 samples (Table 4). One of the most characteristic pigments for brown macroalgae, fucoxanthin, a carotenoid with great antioxidant and anti-inflammatory properties [49], was also found in both algae (Table 4). Ktari et al. [50] investigated the fucoxanthin content in six Dictyotales after overnight maceration in methanol and found almost 2- and 3.6-fold lower concentrations for *D. dichotoma* and *D. fasciola* when compared to this research. This discrepancy is possibly due to differences in the extraction procedure. 

After the determination of protein content, a detailed amino acid profile was evaluated, since they are the main constructive components of proteins, precursors of nucleic acid biosynthesis, and are considered to be pharmaceutically active and health-promoting substances [51]. Additionally, studies showed that a diet containing functional amino acids (glutamine, arginine, glutamate, leucine, and proline) may be involved in metabolism regulation [52]. In biological samples, proteins are usually hydrolyzed with 6 M HCl at 110 °C for 24 h, and implementation of this method resulted in the complete conversion of glutamate, aspartate, and cysteine into glutamic acid, aspartic acid, and cystine, respectively. However, the hydrolysis caused the degradation of tryptophan [53]. Additional hydrolysis in an alkalic solution (e.g., 4 M KOH [26]) should be performed for its determination, but this was not the main objective of this research. Marine macroalgae, as a potent source of bioactive compounds, also contain proteins in different amounts (as mentioned above), and consequently different amino acid compositions were observed. Glutamic acid and aspartic acid were the most abundant in both *D. dichotoma* and *D. fasciola* samples (Figure 1), which is in accordance with the already published data [6] where the amino acid profile of *D. dichotoma* revealed similar results for glutamic acid and leucine, while aspartic acid and arginine levels were somewhat lower than in this study. El Shenody et al. [1] also found 17 amino acids in the *D. dichotoma* sample with the highest amounts of glutamic acid, aspartic acid, alanine, and leucine. Additionally, *D. dichotoma* had the highest amount of amino acids when compared to the results for two other brown macroalgae, *Turbinaria decurrens* and *Laurencia obtusa* [1]. In addition, one should observe that the percentage of essential amino acids found in both *D. dichotoma* and *D. fasciola* is similar (around 40%), but it is important to highlight that the total amount of amino acids was 2.5-fold higher for *D. dichotoma*. These results indicate the importance of content evaluation for all species because variance can be found even within the same genus. 

In living cells, fatty acids serve as fuel for metabolism and muscular contractions and play an important role in health, metabolism, and various diseases. Their role in disease prevention and treatment may be positive or negative; for example, saturated fatty acids may increase the risk of cardiovascular diseases, while polyunsaturated fatty acids may have beneficial effects in patients with multiple sclerosis [54]. Bogaert et al. [6] reported that among brown macroalgae, the genus Dictyota has the highest fatty acid content with a low ω6/ω3 ratio (0.3 to 3.9) and a large amount of ω3 fatty acids. The amount of saturated fatty acids (SFAs) varied from 26 to 56.9%, monounsaturated (MUFAs) from 11.3 to 22.2%, while polyunsaturated fatty acids (PUFAs) varied from 18.3 to 58% for the genus *Dictyota* [6]. This is in agreement with the obtained results in this study, except for the amount of MUFAs, which were herein 1.3 to 2.5-fold higher. El-Shenody et al. [1] reported relatively high total lipid content in *D. dichotoma* (7.5 ± 0.24%) with predominant SFAs. As can be seen from Table 2, among the saturated fatty acids, the highest content was obtained for myristic acid (C14:0) in *D. dichotoma* and palmitic acid (c16:0) in *D. fasciola,* which agrees with the published data [55], where the most abundant SFAs in the *D. bartayresii* were also C16:0 and C14:0. Other research reported the presence of palmitic, linoleic, and arachidonic acid in *D. dichotoma* samples [56], which is also in agreement with the results obtained in our study (Table 2). There were 21 fatty acids in total found in *D. dichotoma*, and 15 of them were found in the *D. fasciola* sample. A statistically significant difference among tested samples was observed for palmitic acid (C16:0), oleic acid (C18:1 (c, t9)), and docosadienoic acid (C22:2 (c13, c16)), which were higher in the *D. fasciola* sample (*p* < 0.0001). When compared to the literature [57], the ω6/ω3 ratio of both Dictyota species a was around 5-fold higher, and a desired ratio of ω6 and ω3 (between 1.5 and 3) [58] was not achieved. However, the presence of essential fatty acids, namely linoleic acid (C18:2, ω6) in both samples, and α-linoleic acid (18:3n-3), γ-linolenic acid (C18:3, ω3), and the eicosanoid precursors arachidonic acid (C20:4, ω6) in the *D. dichotoma* sample indicate the potency of the Dictyota genus. In addition, it is noteworthy to mention that, unlike other fatty acids, stearidonic acid was not found in extracts by GC-FID (Table 2), but was detected by HPLC-MS/MS, which can be explained by its predominant presence in the composition of polar glycolipids that are preferably ionized under ESI conditions.

Further, the non-targeted screening using the HPLC-HRMS technique revealed the presence of numerous compounds (see Table 3), among which the identification of different diterpenoids in Dictyota samples represented the biggest challenge. The identified dolastane diterpenoid, dichotenone, is already known as a *D. dichotoma* and *D. cervicornis* secondary metabolite, which was previously isolated and identified by NMR spectroscopy [59,60]. Mass spectrometry identification of the second compound (amijitrienol) was a more difficult task, since its elemental composition can correspond to the structures of both diterpenoid dolastane and a widespread natural compound retinol. Despite the similarity of their tandem mass spectra, the unambiguous choice in favor of amijitrienol was made based on the UV spectrum, in which absorption of the conjugated system of retinol (~300 nm) was not observed. Being similar in structure to dichotenone and differing from it in the absence of a carbonyl group, amijitrienol was described in the literature as a component of *D. linearis* extracts [61].

In the evaluation of the possible usage of the bioactive extracts or fractions obtained from natural sources, it is crucial to test their potential toxicity [62]. The observed difference in toxicity between the F3 and F4 fractions of *D. fasciola* and *D. dichotoma* points to their different chemical composition. When compared, F3 fractions contained fatty acid amides, namely erucamide and 9-octadecenamide, which could be associated with a negative impact on zebrafish embryonic development. Similar effects were observed in the recent studies on macroalgae *Ericaria crinita*, *E. amentacea* [32], and *Dasycladus vermicularis* [63]. Furthermore, the detected higher toxicity of the *D. dichotoma* F3 fraction in comparison to the *D. fasciola* F3 fraction could be ascribed to higher amounts of terpenoids detected by HPLC-HRMS. The toxicity of terpenoids is not yet fully understood, as they can exhibit both positive [54] and negative [64] effects. However, some recent studies [65] point out the need for toxicity testing of individual terpenoid molecules, especially monoterpenes. Further studies regarding the isolation of terpenoids from algae samples are encouraged.

Complex fractions can contain various molecules that exhibit antioxidant activity by several mechanisms of action [58,66]. In this study, four assays were employed to investigate the antioxidant activity of fractions. By implementing the DPPH assay, 1.6-fold higher activity was noticed for *D. dichotoma* F3 than for the *D. fasciola* F3 fraction. Previous research reported the low antioxidant potential for *D. dichotoma* and *D. spiralis* methanolic extracts, with an inhibition of 4.01% and 9.36%, respectively, at a concentration of 5 mg/mL [5]. Low DPPH activity was also reported for ethanolic extracts of brown macroalgae *D. dichotoma* and *Padina Pavonica* [20]. It has been reported that fucoxanthin is one of the major pigments in brown macroalgae responsible for antioxidant activity [58] and in both *D. dichotoma* and *D. fasciola* F3 samples, fucoxanthin was one of the detected pigments. Consequently, we may assume that fucoxanthin is one of the components responsible for the observed antioxidant activity of F3 fractions. Additionally, a determined diterpenoid only in the *D. dichotoma* F3 fraction, amijitrienol (Table 3), is reported to have antioxidant and anti-tumor activity [54,56,58], while loliolide, present in the F3 fractions of both *D. dichotoma* and *D. fasciola* was reported to exhibit strong antioxidant activity [67]. By implementing ABTS assay, the highest inhibition was observed for *D. fasciola* F4, followed by the *D. dichotoma* F3 fraction. As can be seen in Table 6, the *D. fasciola* F4 sample had the lowest IC_50_ value among tested samples, followed by *D. dichotoma* F3, which was 1.7-fold higher.

Interestingly, the *D. fasciola* F3 sample had the highest IC_50_ value, indicating the lowest antioxidant activity among the tested samples, and its value was 3.7-fold higher than the *D. fasciola* F4 sample and 2.2-fold higher than the *D. dichotoma* F3 sample. Non-targeted analysis revealed several components that were most pronounced in *D. fasciola* F4, including pigment pheophytin b and ceramides known for their antioxidant, anti-aging, anti-inflammatory, and immune regulatory properties [68]. Compared to the literature, the obtained IC_50_ values for both samples were higher than IC_50_ values of brown macroalga *D. vermicularis* F3 and F4 (8.0-fold higher for *D. dichtoma* F3, and 17.5- and 3.0-fold higher for *D. fasciola* F3 and F4 sample, respectively) [63]. Additionally, a recent study reported lower IC_50_ values for two Ericaria species when compared to our study [32]. However, the obtained results for *D. dichotoma* F3 are in accordance with previous research [69] where *D. dichotoma* methanolic extract showed inhibition of 44.8% at 2 mg/mL. In the same research, dichloromethane extraction of the *D. dichotoma* sample was performed with a 2.4-fold higher inhibition when compared to our results for *D. dichotoma* F4 [69]. ORAC results revealed significantly higher antioxidant activity for both *D. dichotoma* and *D. fasciola* F3 fractions than for F4 fractions of samples. The last implemented method was FRAP, and the results revealed approximately two-fold higher activity for both F3 and F4 *D. dichotoma* fractions than those of *D. fasciola*, indicating electron transfer as one of the main mechanisms of action in these samples [70]. Multiple regression analysis (Pearson’s correlation coefficient) was used to evaluate the correlation between all used spectrophotometric methods (ABTS, DPPH, ORAC, and FRAP) [71]. A higher positive correlation was only observed between DPPH and ORAC (*r* = 0.85), while the medium negative correlation between FRAP versus DPPH and ABTS was found with Pearson’s correlation coefficients of −0.40 and −0.34, respectively. Additionally, a medium positive correlation between ABTS and DPPH (*r* = 0.39) was found. However, the significance level was not sufficient enough, suggesting that the used methods correspond to different antioxidant modes of action and are a good indicator of the diverse activity of complex fractions. 

Regarding the antimicrobial activity, partial or complete inhibitory effects of fractions of *D. dichotoma* and *D. fasciola* against both *E. coli* and *S. aureus* was observed. Previously, methanolic, dichloromethane, and hexane extracts of *D. dichotoma* obtained from the Aegean coast were tested for antimicrobial properties using the disk diffusion method [69]. Dichloromethane extracts showed the best inhibitory activity against Gram-positive bacteria. In two additional studies, the activity of ethanolic and aqueous extracts of *D. dichotoma* was also tested using the disk diffusion method and showed activity against both Gram-positive and Gram-negative bacteria [72,73]. Several studies have investigated the antimicrobial activity of *D. dichotoma* using the microdilution method with different types of extracts. In a study by Aydin, the MIC was achieved with DMSO extracts at high concentrations for most of the tested bacteria (>12.5 mg/mL), except for *B. cereus* and *P. aeruginosa*, surprisingly [74]. In our study, the highest tested concentrations of F4 fractions (1.5 mg/mL) showed only partial inhibition of the tested Gram-positive and Gram-negative strains. In contrast, the MIC was obtained for the F3 methanolic fractions tested in *S. aureus*. Gram-positive bacteria were more sensitive to the F3 algal fractions than Gram-negative bacteria, probably due to the specific properties of the cell wall of this group of bacteria. Interestingly, Kosanić et al. [75] tested acetone extracts of *D. dichotoma* with the microdilution method and showed different MICs against different microorganisms at concentrations ranging from 0.156 to 2.5 mg/L. The place and time of sampling, as well as the extraction method and solvent, probably contribute to the variations obtained in the different studies. In conclusion, similar EC_50_ values were obtained in our study for both *D. dichotoma* and *D. fasciola*, suggesting that both species have similar antimicrobial properties and exhibit high efficiency in treating infections caused by Gram-positive pathogens.

The search for compounds with antiaging activity is a hot topic in the natural products field, especially concerning the protection and conservation of the skin and extracellular matrix structure [76]. In our research, the *D. fasciola* F4 sample showed 5.2-fold higher anti-collagenase activity than the *D. fasciola* F3 sample and 13.4-fold higher than the *D. dichotoma* F4 sample at the concentration of 0.5 mg/mL (Figure 4a). Results from this study indicate that three fractions (F3 and F4 of *D. fasciola* and F4 of *D. dichotoma*) have great inhibitory activity against collagenase. This is in agreement with the reported research, where among various fractionated extracts of the brown macroalgae *Dictyota* sp., 8 out of 30 extracts presented 50% inhibition or more at 250 µg/mL, one reaching 81.7%, which consequently indicates a great potential for further development of anti-aging skin products and applications in cosmetic and cosmeceutical industries [76]. According to Mansauda et al. [77], 54.5% collagenase inhibition on the extract of *S. plagiophylloides* is attributed to the high concentration of extracted phloroglucinols.

The damaging effect of the sun (sunburns and hyperpigmentation of human skin) can further lead to several pathological conditions [78]. Melanogenesis and skin pigmentation represent one of the most important photoprotective factors in response to ultraviolet radiation [79]. The main pigment melanin is produced by melanocytes through melanogenesis, while the enzyme tyrosinase plays an important role in the biosynthesis of melanin pigment in the skin [78]. Therefore, the identification and isolation of new tyrosinase inhibitors to control its activity are currently in focus. When compared to the literature, different activity was obtained for brown algae. Methanolic extract (80%) of *P. boergesenii* showed the highest diphenolase activity among 17 species with 36.68% inhibition (250 µg/mL), while Cystoseira and Sargassum species showed inhibition from 7 to 20% [80]. Algae that can inhibit both monophenolase and diphenolase tyrosinase activities are more potent inhibitors [80]. However, both Dictyota species in this study exerted low or even no monophenolase activity, while the diphenolase activity of *D. dihotoma* F3 fraction was almost 30%. Further, the *D. fasciola* F3 fraction had two-fold lower activity along with the *D. dihotoma* F4 fraction, while the *D. fasciola* F4 fraction had significantly (*p* < 0.05) the lowest activity (Figure 4b). Previously reported research associated high antityrosinase activity (both mono- and diphenolase) with specifically isolated phlorotannins from brown alga *E. stolonifera* [81]. Conversely, in this study, no phlorotannins could be identified, but the presence of fucoxanthin and terpene compounds, especially in F3 fractions, can explain the observed anti-melanogenic effect [16]. Interestingly, the determined loliolide (Table 3) was reported to have exceptional photoprotective, anti-inflammatory, anti-wrinkling, and antiaging activities [67], which can also contribute to the observed inhibition.

The α-amylase and pancreatic lipase are digestive enzymes that are effective targets in obesity and type II diabetes treatment [82]. The α-amylase is the secretory product of salivary glands and pancreas, and also one of the most important digestive enzymes in carbohydrate breakdown [83]. Because of the possible undesirable side effects of some antidiabetic drugs that act by inhibiting α-amylase, there is an increased need for natural α-amylase inhibitors [84]. On the other hand, the inhibition of pancreatic lipase decreases the digestion of triglycerides from the food which leads to monoacylglycerols and free fatty acid reduction in the intestinal lumen [82], which is important in some disease treatments (i.e., obesity). As can be seen in Figure 5a, the *D. dichotoma* F3 fraction had 2.1-fold higher inhibitory activity than the *D. fasciola* F3 fraction. In contrast, the *D. fasciola* F4 fraction resulted in 1.8-fold higher inhibitory activity than the *D. dichotoma* F4 sample. The obtained results for both the *D. dichotoma* and *D. fasciola* F3 fractions are much higher than those previously reported on the same concentrations for several brown macroalgae methanolic extracts [85]. The inhibitory activity against α-amylase was also observed for the fucoxanthin fraction and methanolic extract obtained from brown seaweeds *S. siliquosum* and *S. polycystum* [85]. The inhibitory activity of methanolic fractions of *D. dichotoma* and *D. fasciola* can also be ascribed to the presence of fucoxanthin. Results regarding pancreatic lipase inhibition revealed that *D. dichotoma* and *D. fasciola* have shown good inhibitory rates compared to the positive control, orlistat. To the best of our knowledge, scarce literature data are available about the lipase inhibitory potential of extracts of the genus Dictyota. Based on the available data, most of the studies on algae connected the lipase inhibitory activity to the presence of polysaccharides [7,13] and phlorotannins [86]. It is known that brown macroalgae generally contain large amounts of compounds exhibiting lipase-inhibiting activity, such as organic heterocyclic compounds (porphyrin structure) and chlorophylls which can be linked to the chlorophyll derivatives found in our fractions, pheophytin a and b. It was previously demonstrated that fucoxanthin also reduces obesity by altering lipid metabolism [87,88], so in this research, a good pancreatic lipase inhibition, obtained for all fractions, especially F3, can be attributed to its presence. Interestingly, ethanolic extract from *D. fasciola* had no observed inhibitory activity against pancreatic lipase [89], while Biotu et al. [90] covered the research of methanolic extracts from 54 marine algae species and brown algae displayed relatively high inhibition potential toward pancreatic lipase activity (>50%). 

One of the most known diseases directly connected with the deficiency of the neurotransmitter acetylcholine in the brain is Alzheimer’s disease. A promising treatment approach emphasizes the inhibition of the acetylcholinesterase enzyme (AChE). It has been previously reported that a variety of plants possess acetylcholinesterase inhibitory activity [91], but inhibitory activity of several marine algae species has also been reported [92]. Furthermore, Pangestuti and Kim [91], in research from 2011, showed that compounds derived from macroalgae may improve memory and learning functions in various neurodegenerative conditions. The results obtained in our research were similar for both *D. dichotoma* and the *D. fasciola* sample. Previously, the methanolic extract of *D. dichotoma* was tested for its acetylcholine esterase inhibitory effect, which was comparable to that of the F3 fraction in our study, and the results were 17-fold lower than ours [93]. Alghazwi et al. [12] summarized the macroalgae-derived compounds that are targeting cholinesterase activity and, in our research, loliolide and fucoxanthin have been found in both tested samples, while α-linolenic acid was found only in *D. dichotoma* sample, as molecules with enzyme inhibitory effects [10,94]. Aly et al. [95] reported the presence of α-linolenic acid and cystoseirol monoacetate, compounds with good binding affinities to the AChE enzyme [95], in the methanolic fraction of several Cystoseira species, while the same compounds were also determined in this research for the *D. dichotoma* F3 fraction. On the other hand, ethanolic extracts of *D. dichotoma* did not show inhibitory effects on acetylcholine esterase [94]. In other research, the most active extract with AChE inhibitory activity was the methanolic extract of *Dictyota humifusa* [96]. When comparing results, two Dictyota species in this study showed moderate, but similar acetylcholinesterase inhibitory activity, which is connected to the detected and identified compounds in both samples. 

## 5. Conclusions

In this study, we determined macronutrient content (total proteins, amino acids, total carbohydrates, pigments, and fatty acid profile), including antioxidant, antimicrobial, embryotoxic, and enzyme inhibition potential of non-polar factions from two brown macroalgae, *D. dichotoma* and *D. fasciola* from the Adriatic Sea, together with non-targeted HPLC-MS analysis of the compounds present in the samples. Results of chemical and antioxidant analysis revealed that the compounds and their modes of action acted synergistically in the samples, which impacted the antioxidant response. Additionally, the differences in obtained protein, amino acid, pigment, and fatty acid composition emphasize the importance of macroalgal research within the same species because different composition causes different activity. Moderate anti-collagenase and anti-tyrosinase activity have shown the promising potential of Dictyota species as a source of bioactive ingredients for dermatological applications and research of novel cosmetics. Additionally, the obtained results for α-amylase, pancreatic lipase, and acetylcholinesterase inhibition are significantly higher than previously reported, making these macroalgae interesting natural sources of enzyme inhibitors. However, further studies are necessary to investigate the influence of harvesting season, location, and distribution between the same species. Additionally, additional studies should be encouraged, not just for these two species, but for macroalgae in general because of their unique properties which make them great candidates for nutraceutical applications. All obtained results indicate that both *D. dichotoma* and *D. fasciola* have the potential for usage in diverse industries such as food, cosmetics, pharmaceutical, and others.

## Figures and Tables

**Table 1:** Total protein, carbohydrate, and chlorophyll content in two brown seaweeds from the Dictyota family. All results are expressed as replicates.

Phytochemical Content	*D. dichotoma*	*D. fasciola*
Total proteins, %	7.420 ± 0.753	3.301 ± 0.004
Total carbohydrates, mg/g	758.469 ± 3.034	712.988 ± 2.852
Chlorophyll a, mg/g DW *	3.091 ± 0.004	0.356 ± 0.001
Chlorophyll b, mg/g DW *	0.382 ± 0.004	0.005 ± 0.001
Carotenoids, mg/g DW *	0.772 ± 0.001	0.131 ± 0.002
Pheophytin a, mg/g DW *	4.519 ± 0.013	0.627 ± 0.002
Pheophytin b, mg/g DW *	2.157 ± 0.006	0.299 ± 0.001

* DW—dry weight.

**Table 2:** Fatty acids composition of *D. dichotoma* and *D. fasciola* determined by GC-FID analysis.

Lipid Numbers	Fatty Acid	*D. dichotoma*	*D. fasciola*
		Av ± SD *, %	
C4:0	Butyric acid	-	0.94 ± 0.28
C8:0	Caprylic acid	-	1.09 ± 0.36
C10:0	Capric acid	2.70 ± 0.02	1.41 ± 0.37
C12:0	Lauric acid	2.01 ± 0.03	-
C14:0	Myristic acid	16.68 ± 0.23	10.05 ± 0.6
C14:1	Myristoleic acid	4.50 ± 0.37	-
C15:0	Pentadecylic acid	2.54 ± 0.04	0.90 ± 0.12
C15:1	Pentadecenoic acid	1.24 ± 0.01	-
C16:0	Palmitic acid	4.80 ± 0.04	27.49 ± 1.72
C16:1(c9)	Palmitoleic acid	11.65 ± 0.13	3.02 ± 0.42
C17:1 (c10)	Heptadecenoic acid	1.26 ± 0.03	-
C18:0	Stearic acid	7.54 ± 0.02	4.19 ± 0.93
C18:1 (c9, t9)	Oleic acid	6.81 ± 0.07	24.37 ± 0.76
C18:2 (c9, c12)	Linoleic acid, ω6	3.77 ± 0.14	3.13 ± 0.16
C18:2 (t9, t12)	Linolelaidic acid	3.74 ± 0.12	-
C18:3 (c6, c9, c12)	γ-Linolenic acid, ω6	4.44 ± 0.07	5.13 ± 0.62
C20:1 (c11)	Gondoic acid	1.55 ± 0.03	0.65 ± 0.19
C18:3 (c9, c12, c15)	α-Linolenic acid, ω3	1.25 ± 0.04	-
C21:0	Heneicosylic acid	1.37 ± 0.06	-
C20:2 (c11, c14)	Eicosadienoic acid, ω6	1.65 ± 0.03	-
C20:3 (c8, c11, c14)	Dihomo-γ-linolenic acid, ω6	14.92 ± 0.13	5.93 ± 0.43
C20:3 (c11, c14, c17)	Eicosatrienoic acid, ω3	3.57 ± 0.04	2.88 ± 0.80
C20:4 (c5, c8, c11, c14)	Arachidonic acid, ω6	1.36 ± 0.00	-
C22:2 (c13, c16)	Docosadienoic acid, ω6	-	10.6 ± 1.01
Saturated fatty acids (SFA, %)		38.99 ± 0.45	45.90 ± 3.90
Monounsaturated fatty acids (MUFA, %)		28.95 ± 1.67	28.17 ± 1.18
Polyunsaturated fatty acids (PUFA, %)		29.57 ± 0.43	27.23 ± 3.03

* Av—an average area of three replicates expressed in percentage (%) with standard deviation (SD).

**Table 3:** Major non-polar molecules identified in *D. dichotoma* and *D. fasciola* by LC-MS/MS analysis.

No.	Proposed Compound	RT, min	Elemental Composition	*m/z*	*D. dichotoma*		*D. fasciola*	
					F3	F4	F3	F4
1.	Loliolide	9.0	C_11_H_16_O_3_	197.1172	908,277	-	374,832	-
2.	Diterpenoid *	12.8	C_20_H_30_O_3_	319.2271 [M + H − H_2_O]^+^	360,525	-	-	-
3.	Cystoseirol monoacetate	13.1–14.2	C_22_H_36_O_5_	381.2636	595,000	-	-	-
4.	Dichotenone or/and Dichotenone A	16.6 17.1	C_20_H_30_O_4_	352.2482 [M + NH_4_]^+^	1,012,627 1,083,711	-	-	-
5.	(3S)-3beta-[(1R,4R)-1,5-Dimethyl-4-hydroxy-5-hexenyl]-7beta-hydroxy-7abeta-methyl-2,3,3a,6,7,7a-hexahydro-1H-indene-3abeta,4-dicarbaldehyde	17.2	C_20_H_30_O_4_	335.2219	575,382	26,226	708,539	155,973
6.	DGMG (18:4) (first isomer)	18.1	C_33_H_54_O_14_	692.3848 [M + NH_4_]^+^	227,393	-	42,432	-
7.	Dictyotatriol A	18.3	C_20_H_34_O_3_	323.2573	59,323	1814	92,362	10,624
8.	DGMG (18:4) (second isomer)	18.5	C_33_H_54_O_14_	692.3845 [M + NH_4_]^+^	291,683	-	22,833	-
9.	DGMG (16:1) (first isomer)	18.9	C_31_H_56_O_14_	670.4013 [M + NH_4_]^+^	115,032	-	14,063	-
10.	Diterpenoid*	19.0	C_22_H_38_O_5_	383.2796	168,287	-	-	-
11.	DGMG (16:1) (second isomer)	19.3	C_31_H_56_O_14_	670.4001 [M + NH_4_]^+^	154,546	-	11,035	-
12.	5-Methoxy-3-tridecyl-1,2,4-benzenetriol	19.5	C_20_H_34_O_4_	339.2530	103,854	-	282,539	-
13.	MGMG (18:4) (first isomer)	19.6	C_27_H_44_O_9_	530.3325 [M + NH_4_]^+^	967,875	-	161,929	-
14.	MGMG (18:4) (second isomer)	19.9	C_27_H_44_O_9_	530.3325 [M + NH_4_]^+^	1,295,791	-	171,568	-
15.	Amijitrienol	21.3	C_20_H_30_O	287.2369	5,968,809	-	-	-
16.	(4R,4aR,7S,8Z,10S,12aR)-10-Hydroxy-4-isopropyl-1-methyl-3,4,4a,5,6,7,10,11,12,12a-decahydrobenzo [10] annulen-7-yl acetate	21.6	C_20_H_32_O_3_	321.2424	-	176,067	266,152	1,022,611
17.	triacetoxy-18-hydroxy-2,7-dolabelladiene	21.9	C_26_H_40_O_7_	482.3112 [M + NH_4_]^+^	21,974	3838	5,264,917	35,357
18.	5,6,18-triacetoxy-hydroxy-dolabelladiene	22.7	C_26_H_40_O_7_	482.3109 [M + NH_4_]^+^	16,480	694	3,714,070	11,212
19.	Diacetylated diterpenoid *^,1^	23.8	C_24_H_38_O_5_	407.2785	-	-	794,540	-
20.	5,6,10,18-tetracetoxy-2,7-dolabelladiene	24.2	C_28_H_42_O_8_	524.3212 [M + NH_4_]^+^	22,124	4916	6,080,089	37,270
21.	(3β,7α,11α)-7,9,11-Trihydroxycholest-8(14)-en-3-yl acetate	24.4	C_29_H_48_O_5_	477.3575	18,836	794,709	10,704	3,215,228
22.	18-acetoxy-10-hydroxy-2,7-dolabelladiene	24.7	C_22_H_36_O_3_	366.2995 [M + NH_4_]^+^	331,590	-	-	-
23.	9-Octadecenamide	25.80	C_18_H_35_NO	282.2791	894,479	24,674	2,639,470	97,376
24.	5-Hydroxycholesta-7,9(11),22-trien-3-yl acetate	26.1	C_29_H_44_O_3_	441.3363	27,000	83,051	29,000	524,629
25.	Glyceryl stearate	26.6	C_21_H_42_O_4_	359.3156	968,035	13,726	1,560,128	49,999
26.	DGDG (20:5/18:4)	26.9	C_53_H_82_O_15_	976.5992 [M + NH_4_]^+^	170,626	-	384,283	-
27.	Oleic acid	26.9	C_18_H_34_O_2_	283.2632	279,635	-	453,666	-
28.	Diacetylated diterpenoid *^,2^	27.2	C_24_H_40_O_8_	474.3061 [M + NH_4_]^+^	7600	-	252,949	-
29.	6-Hydroxystigmasta-4,22-dien-3-one	27.4	C_29_H_46_O_2_	427.3571	83,000	874,722	151,000	3,698,835
30.	Fucoxanthin	27.8	C_42_H_58_O_6_	659.4306	28,519	-	128,869	-
31.	10-Hydroxypheophorbide a	28.0	C_35_H_36_N_4_O_6_	609.2708	806,000	-	167,000	-
32.	MGDG (18:4/18:4)	28.4	C_45_H_70_O_10_	788.5308 [M + NH_4_]^+^	581,218	-	1,030,644	-
33.	Erucamide	29.0	C_22_H_43_NO	338.3417	1,681,231	709,883	5,956,202	1,523,221
34.	Pheophorbide A	29.5	C_35_H_36_N_4_O_5_	593.2758	1300	41,997	335,625	343,127
35.	DGDG (16:0/18:1)	29.5	C_49_H_90_O_15_	936.6601 [M + NH_4_]^+^	61,910	11,070	130,962	73,980
36.	Neutral glycosphingolipid *^,3^	30.3	C_44_H_83_NO_9_	770.6141	2836	60,566	7300	567,651
37.	Neutral glycosphingolipid *^,4^	30.5	C_46_H_85_NO_9_	796.6297	-	58,843	-	744,495
38.	MGDG (18:1/14:0)	30.6	C_41_H_76_O_10_	746.5767 [M + NH_4_]^+^	304,323	79,663	790,661	393,564
39.	MGDG (18:2/16:0)	30.8	C_43_H_78_O_10_	772.5926 [M + NH_4_]^+^	140,870	12,251	304,491	103,172
40.	alpha-Tocomonoenol	31.5	C_29_H_48_O_2_	429.3727	460,197	89,913	712,772	526,633
41.	MGDG (18:1/16:0)	31.6	C_43_H_80_O_10_	774.6078 [M + NH_4_]^+^	79,980	147,000	586,000	672,000
42.	MGDG (16:0/18:1)	31.7	C_43_H_80_O_10_	774.6090 [M + NH_4_]^+^	65,002	145,985	564,653	617,779
43.	Methyl (22S,23S)-12-ethyl-3-hydroxy-17-(1-hydroxyethylidene)-13,18,22,27-tetramethyl-5-oxo-23-[3-oxo-3-[(E,7R,11R)-3,7,11,15-tetramethylhexadec-2-enoxy]propyl]-4-oxa-8,24,25,26-tetrazahexacyclo [19.2.1.16,9.111,14.116,19.02,7]heptacosa-1,6(27),7,9,11(26),12,14,16(25),18,20-decaene-3-carboxylate	37.0	C_55_H_74_N_4_O_8_	919.5579	18,800	375,787	12,156	3,904,305
44.	Methyl (3R,10Z,14Z,20Z,22S,23S)-12- ethyl-3-hydroxy-13,18,22,27- tetramethyl-5-oxo-23-(3-oxo-3- {[(2E,7R,11R)-3,7,11,15-tetramethyl-2- hexadecen-1-yl]oxy}propyl)-17-vinyl-4- oxa-8,24,25,26-tetraazahexacyclo[19.2.1.16,9.111,14.116,19.02,7]heptacosa- 1(24),2(7),6(27),8,10,12,14,16,18,20- decaene-3-carboxylate	37.3	C_55_H_74_N_4_O_7_	903.5630	31,553	5,903,021	40,705	44,694,373
45.	Pheophytin b	37.6	C_55_H_72_N_4_O_6_	885.5525	3800	227,006	28,166	1,210,828
46.	Hydroxypheophytin a	37.6	C_55_H_74_N_4_O_6_	887.5681	427,334	712,551	264,658	6,721,477
47.	Chlorophyll derivative *	37.7	C_55_H_74_N_4_O_9_	935.5529	4800	195,600	7000	878,000
48.	Pheophytin a	38.1	C_55_H_74_N_4_O_5_	871.5732	1948	8,833,018	888,174	77,422,991

* unidentified; ^1^ likely, (6b,7b,13R)-6,7-Diacetoxy-8,14-labdadiene-13-ol or similar structure; ^2^ likely, klyxumine A or a similar structure; ^3^ likely, N-(2R-hydroxyicosanoyl)-1-β-glucosyl-4E,8E-octadecasphingadienine; ^4^ likely, Ophidiacerebroside B.

**Table 4:** The pigment composition of *D. dichotoma* and *D. fasciola* determined by HPLC. The results are presented as the average area of three replicates expressed in mg/g of a dry fraction with standard deviation. Values with the same letter within a row are significantly different (*p* < 0.05).

Pigment	*D. dichotoma*		*D. fasciola*	
	F3	F4	F3	F4
Fucoxanthin	5.01 ± 0.63 ^a,b^	0.35 ± 0.01 ^a^	3.15 ± 0.96 ^b^	n.d.
Lutein	n.d.	n.d.	0.27 ± 0.06	n.d.
Chlorophyll a	n.d.	1.31 ± 0.21 ^c^	n.d.	0.44 ± 0.04 ^c^

n.d.—not detected.

**Table 5:** Acute toxicity of *D. fasciola* and *D. dichotoma* fractions (F3 and F4) obtained upon 96 h of zebrafish *D. rerio* exposure.

(a) Sample		LC_50_ Value, mg/mL	Confidence Interval	R^2^ Value	Hillslope
*D. dichotoma*	F3	0.33 × 10^−3^	0.33 × 10^−3^–0.34×10^−3^	0.989	36.19
	F4	>0.01 *	n.d.	n.d.	n.d.
*D. fasciola*	F3	0.03	0.03–0.03	0.995	11.32
	F4	>0.19 *	n.d.	n.d.	n.d.
(b) Sample		EC_50_ Value, mg/mL	Confidence Interval	R^2^ Value	Hillslope
*D. dichotoma*	F3	0.31 × 10^−3^	0.31 × 10^−3^–0.31 × 10^−3^	0.979	64.70
	F4	>0.01 *	n.d.	n.d.	n.d.
*D. fasciola*	F3	0.02	0.02–0.03	0.984	7.70
	F4	>0.19 *	n.d.	n.d.	n.d.

* maximal effect not reached. n.d. = not determined.

**Table 6:** Calculated half-maximal inhibitory concentrations (IC_50_) with the presented confidence intervals, R^2,^ and Hillslope values obtained using the ABTS assay (*n* = 3).

Sample		IC_50_ Value, mg/mL	Confidence Interval	R^2^ Value	Hillslope
*D. dichotoma*	F3	3.974	3.624–4.438	0.996	1.296
	F4	n.d.	-	-	-
*D. fasciola*	F3	6.167	5.319–7.587	0.974	1.513
	F4	2.372	1.913–3.185	0.974	1.526

n.d.—not determined.

## Data Availability

Not applicable.
